# In vitro and patient studies with platelets to explore off‐target cardiovascular effects of integrase inhibitors

**DOI:** 10.1111/hiv.13738

**Published:** 2024-11-15

**Authors:** R. Keniyopoullos, A. A. Khawaja, M. Boffito, M. Emerson

**Affiliations:** ^1^ National Heart and Lung Institute Imperial College London London UK; ^2^ Department of Infectious Disease Imperial College London London UK; ^3^ Chelsea and Westminster Hospital, NHS Foundation Trust London UK

**Keywords:** antiretroviral, cardiovascular, integrase inhibitors, pharmacology, platelets

## Abstract

**Introduction:**

People with HIV currently face a tenfold higher risk of developing cardiovascular disease (CVD) than those without HIV. Studies have shown various off‐target effects of antiretroviral treatment (ART) on the cardiovascular system, but little is known about the effects of currently used integrase strand transfer inhibitors (INSTIs) on platelets. Platelet activation is associated with increased CVD, thrombus formation, and release of proinflammatory mediators, so exploring platelet effects from currently prescribed ART may contribute to the understanding of CVD etiopathogenesis in people with HIV.

**Methods:**

We aimed to identify potential effects of INSTIs on platelet aggregation and activation markers from individuals without HIV after in vitro treatment with clinically relevant drug concentrations. We used bictegravir (BIC) and dolutegravir (DTG) individually or in the therapeutic drug combinations BIC/emtricitabine (FTC)/tenofovir alafenamide fumarate (TAF) or DTG/lamivudine (3TC). Additionally, we conducted a pilot study to compare platelet activity profiles from people with HIV on BIC/FTC/TAF and DTG/3TC.

**Results:**

Changes to in vitro platelet aggregation responses upon exposure to different INSTIs were observed both upon individual drug application and when using therapeutic combinations. However, these effects were not reflected in flow‐cytometric evaluation of platelet degranulation. A pilot study in eight people with HIV and eight without HIV revealed no significant effects but established protocols for future patient studies.

**Conclusion:**

There is currently no consistent evidence of an effect of INSTIs on platelet activation. Further study is warranted, focusing on models with more pathophysiological relevance, including extensive studies in people with HIV.

## INTRODUCTION

Effective antiretroviral treatment (ART) means that people with HIV are expected to live into older age and acquire a range of comorbidities associated with ageing. Cardiovascular disease (CVD) is the most prevalent comorbidity observed, despite the efficacy of ART in controlling HIV [1], and both HIV and certain ART regimens have been shown to be independent risk factors for CVD [2, 3]. Both clinical cardiovascular events, including myocardial infarction (MI) [4], and subclinical cardiovascular damage are elevated in people with HIV [5]. Platelets are key components of CVD, and they interact directly with HIV and HIV‐encoded proteins, leading to abnormal platelet function [6] that persists during effective therapy [7]. Furthermore, there are established links between inflammation and platelets, despite control of HIV replication [8], and multiple reports of persistently heightened platelet function despite effective therapy [7, 9, 10]. There is a consensus, therefore, that platelet function is modified in people with HIV, although much remains unknown about exactly how platelet function is different and the causes of altered platelet function in people with HIV. Whether or how altered platelet function in the context of HIV links mechanistically with CVD also remains unknown.

There is substantial evidence that certain antiretroviral drugs are associated with increased MI in people with HIV [11] and that they affect platelet function [9, 10]. The most extensively studied drug from a cardiovascular risk perspective is the nucleotide reverse transcriptase inhibitor (NRTI) abacavir sulphate (ABC). ABC was shown to have no direct effect on platelet activation in vitro [12], although we subsequently showed that elevated platelet responses in vivo result from ABC interruption of endothelial‐dependent inhibition of platelet activation in human platelets and a mouse model of platelet thromboembolism [13]. ABC also drives a proinflammatory and thrombotic endothelial phenotype and increases platelet‐endothelial crosstalk [14]. More recently, ABC has been shown to affect leukocytes, specifically leukocyte–platelet crosstalk [15], indicating multiple ABC‐associated effects that could drive cardiovascular risk. In patient studies, switching from ABC to tenofovir‐based therapies increased soluble platelet glycoprotein VI levels [16], and adding ABC to regimens caused reversible changes in platelet vasodilator‐stimulated phosphoprotein phosphorylation [17], possibly indicating negative feedback mechanisms in response to an ABC‐associated thrombotic state. Many protease inhibitors also activate platelets [18], and the protease inhibitor ritonavir was shown to enhance platelet aggregation and disrupt lipid mediator production, specifically synthesis of prostaglandin E_2_ and thromboxane [19].

Emerging evidence suggests that modern drugs outside of the NRTI and protease inhibitor classes also affect platelet function [20]. Detailed comparisons of how antiretrovirals in common clinical use, as well as those emerging into clinical practice, affect platelet function will provide better understanding of the role of natural history versus drug effects in driving CVD in people with HIV and will identify potentially avoidable drivers of CVD in people with HIV. Platelet studies in the HIV field are therefore important and must be conducted carefully and with considered interpretation to avoid premature and unsubstantiated links between antiretroviral exposure and CVD being presented.

Modern ART regimens frequently include integrase strand transfer inhibitors (INSTIs) in combination with other classes of drugs such as NRTIs. INSTI‐based therapies are proven to be effective in both treatment‐naïve and treatment‐experienced individuals with HIV [15]. The effects of INSTIs on platelet function and therefore CVD risk is incompletely understood. There are reports of possible associations between INSTI exposure and factors associated with CVD such as metabolic syndrome and weight gain [21, 22]. These studies are far from conclusive, and inconsistencies in findings as well as confounding factors, such as the effects of previous ART exposure, have been highlighted [23]. INSTIs are an important cornerstone of modern, effective HIV control, so detailed understanding of their off‐target effects is warranted alongside further studies to optimize their therapeutic value.

In this study, we assessed the effects of two commonly prescribed INSTIs, dolutegravir (DTG) and bictegravir (BIC), on platelet function using well‐established platelet‐activation assays. We considered drugs given individually and in combinations used in treatment regimens. We compared in vitro studies with patient‐derived data. Although no evidence of an effect of INSTIs on platelets in people with HIV was observed, our study provides justification for further research on this topic as well as insight into the design and interpretation of platelet‐ and other cell‐based pharmacological studies in the context of HIV and ART.

## MATERIALS AND METHODS

### Antiretrovirals and other reagents

BIC and tenofovir alafenamide fumarate (TAF) were supplied by Gilead Sciences (CA, USA); DTG, emtricitabine (FTC), and lamivudine (3TC) were purchased from Selleckchem (TX, USA) and were used at clinical peak plasma concentration (C_max_) values of 13.7, 0.2, 8.8, 8.6, and 6.1 μM, respectively. Adenosine 5′‐diphosphate (ADP; Sigma‐Aldrich) was used at 1–30 μM, thrombin receptor activator for peptide 6 (TRAP‐6; Sigma‐Aldrich) was used at 1–30 μM, and collagen (Takeda) was used at 1–30 μg/mL. ADP and TRAP‐6 were diluted in phosphate‐buffered saline (Cytiva), and collagen was diluted in isotonic glucose (Takeda). Eptifibatide (GSK) was used at 0.6 μg/mL. CD62P:APC and CD63:PE were purchased from BD Biosciences.

### Platelet‐rich plasma (PRP) processing

Whole blood was centrifuged for 15 min at 175 *g* (no brake) to separate platelet‐rich plasma (PRP), 1 mL of which was further centrifuged at 15,800 *g* for 3 min to obtain a platelet‐poor plasma to be used as a blank for light‐transmission aggregometry. In experiments using samples from people without HIV, PRP was incubated with the drug or drug combinations for 25 min before use.

### Light‐transmission aggregometry

Platelets in PRP previously treated with drugs of interest were activated using ADP, TRAP‐6, or collagen in a 96‐well flat‐bottom plate (VWR, Inc.) where absorbance was measured for 15 min at 595 nm. A measurement was made at 37°C every 20 s in a plate reader (Sunrise TECAN), with a shaking step between every recording. Platelet‐poor plasma was used as a blank, and absorbance was used to calculate percentage aggregation. Inhibition of platelet aggregation was achieved by adding 0.6 μg/mL of eptifibatide (an α_ΙΙb_β_3_ inhibitor) per well.

### Platelet activation markers by flow cytometry

Drug‐treated PRP was diluted tenfold in Tyrode's buffer (138 mM NaCl, 2.6 mM KCl, 12 mM NaHCO_3_, 0.2 mM NaHPO_4_, 5.5 mM glucose). CD62P:APC (α‐granule release) and CD63:PE (δ‐granule release) antibodies were used at 1:60 each for every sample, including PRP, Tyrode's buffer, and agonist, which was added last to activate platelets 30 s after initiation of measurement. ADP and TRAP‐6 were used at the final concentration of 10 μM and collagen at 10 μg/mL. Mean fluorescence intensity was measured in Accuri C6 Plus (BD Biosciences), and fold change was calculated for every marker in every sample.

### Statistical analysis

Results passed the Shapiro–Wilk normality test and were analysed using one‐ or two‐way analysis of variance (ANOVA) as indicated in figure legends. Groups were compared using Tukey's multiple comparison in GraphPad (V9). The histograms show the mean average in columns, and error bars indicate the standard error of the mean.

### Recruitment of people with HIV

In total, 16 people with HIV (mean age 53.1 ± 5.9 years) on stable and effective ART with a viral load <50 copies/mL were recruited and consented (Chelsea and Westminster Hospital NHS Foundation Trust) under NHS research ethics committee approval. Subjects with a history of noncompliance, who had changed drug regimen within 1 month of the study, or who were talking antiplatelet medicine were excluded from the study. Whole blood was transported to Imperial College London for PRP processing and light‐transmission aggregometry.

## RESULTS

### Platelet aggregation in vitro shows comparative differences

**FIGURE 1:**
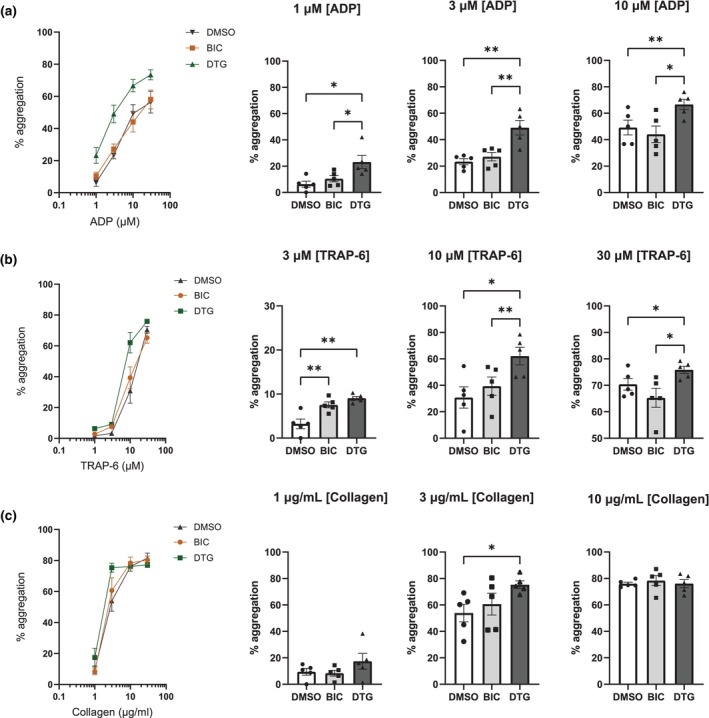
Platelet aggregation upon in vitro treatment with bictegravir (BIC) and dolutegravir (DTG). Panel (a) shows percentage platelet aggregation from *N* = 5 upon exposure to peak plasma concentration (C_max_) of BIC, DTG, or dimethylsulfoxide (DMSO) for 25 min followed by adenosine 5′‐diphosphate (ADP) activation using a range of agonist concentrations from 1 to 30 μM ADP. Similarly, (b) shows thrombin receptor activator for peptide 6 (TRAP‐6)‐evoked platelet aggregation using 1–30 μM TRAP‐6, and (c) shows collagen‐induced aggregation ranging from 1 to 30 μg/mL of collagen. Results were analysed using one‐way analysis of variance, and groups were compared using Tukey's multiple comparison: * *p* < 0.05, ** *p* < 0.01, *** *p* < 0.001.

Platelets in PRP from five people without HIV were activated using a range of agonist concentrations as shown in Figure 1, after 25 min in vitro treatment with 13.7 μM BIC or 8.8 μM DTG. At 1, 3, and 10 μM ADP, dimethylsulfoxide (DMSO)‐treated platelets displayed 6.3%, 23.3%, and 49.2% platelet aggregation; BIC‐treated platelets displayed 10.4%, 27.1%, and 44% platelet aggregation, and DTG‐treated platelets displayed 23.2%, 49%, and 66.6% platelet aggregation, respectively. At 3, 10, and 30 μM TRAP‐6, DMSO‐treated platelets displayed 3.23%, 30.8%, and 70.4% platelet aggregation, BIC‐treated platelets displayed 7.53%, 39.3%, and 65.3% platelet aggregation, and DTG‐treated platelets displayed 9%, 62.1%, and 75.8% platelet aggregation, respectively. Lastly at 1, 3, and 10 μg/mL of collagen, DMSO‐treated platelets displayed 9.4%, 53.9%, and 76% platelet aggregation, BIC‐treated platelets displayed 8.4%, 60.6%, and 78.4% platelet aggregation, and DTG‐treated platelets displayed 17.4%, 75.3%, and 76.1% platelet aggregation, respectively. Comparisons between different treatment groups are displayed in Figure 1.

**FIGURE 2:**
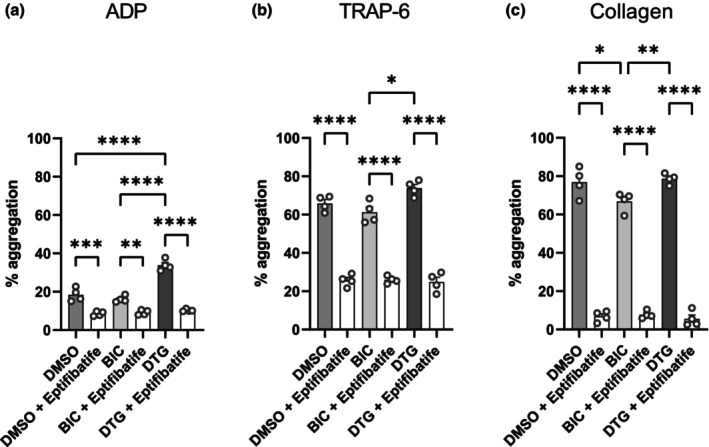
Eptifibatide‐inhibited platelet aggregation after treatment with bictegravir (BIC) or dolutegravir (DTG). Platelets from volunteers without HIV (*N* = 4) were treated with clinically relevant concentrations of BIC, DTG, or vehicle control (dimethylsulfoxide [DMSO]) as well as with 0.6 μg/mL of eptifibatide, a specific α_ΙΙb_β_3_ inhibitor, to block integrin‐mediated platelet aggregation. The graphs show (a) 3 μM adenosine 5′‐diphosphate (ADP), (b) 10 μM thrombin receptor activator for peptide 6 (TRAP‐6), and (c) 3 μg/mL collagen‐evoked platelet aggregation, 25 min after treatment with the drugs ± eptifibatide. Results were analysed using two‐way analysis of variance, and groups were compared using Tukey's multiple comparison: * *p* < 0.05, ** *p* < 0.01, *** *p* < 0.001, **** *p* < 0.0001.

To confirm a true aggregation effect mediated by established platelet‐signalling events rather than a non‐specific effect on light transmission, 0.6 μg/mL of eptifibatide was added to block integrin‐mediated aggregation. A total 3 μM of ADP, 10 μM TRAP‐6, and 10 μg/mL of collagen were added to INSTI‐treated PRP from five volunteers without HIV, with and without eptifibatide, to assess differences in the aggregation profiles. Figure 2 shows that the differences in percentage aggregation between the drug groups (as reported in Figure 1) were reproducible and that, in the presence of eptifibatide, the differences were completely abolished, confirming an α_ΙΙb_β_3_‐mediated effect in every agonist‐mediated activation examined.

### In vitro aggregation effects are sustained when modelling therapeutic drug combinations

**FIGURE 3:**
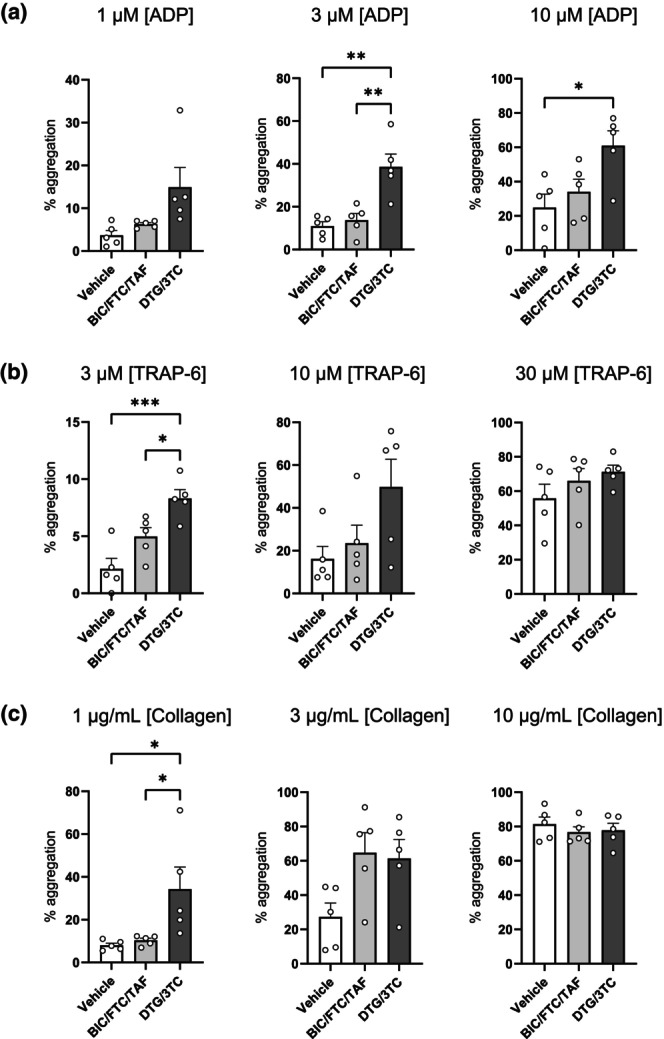
Aggregation profiles of bictegravir (BIC)/emtricitabine (FTC)/tenofovir alafenamide fumarate (TAF)‐ or dolutegravir (DTG)/lamivudine (3TC)‐treated platelets. Top row (a) shows aggregation profiles of adenosine 5′‐diphosphate (ADP)‐evoked platelet activation at increasing agonist concentrations from left to right, after in vitro treatment of platelets from healthy volunteers (*N* = 5) with clinically relevant concentrations of BIC/FTC/TAF, DTG/3TC, or vehicle (dimethylsulfoxide). Row (b) indicates thrombin receptor activator for peptide 6 (TRAP‐6)‐induced and row (c) the collagen‐induced platelet aggregation at increasing concentrations from left to right. Results were statistically analysed using one‐way analysis of variance, and groups were compared using Tukey's multiple comparison: *p < 0.05, **p < 0.01, ***p < 0.001.

To examine a more clinically relevant effect of INSTIs on platelet aggregation, PRP from volunteers without HIV was treated in vitro with common drug combinations DTG and BIC, which are usually prescribed in the combinations DTG/3TC and BIC/FTC/TAF. Figure 3 shows the ADP‐, TRAP‐6‐, and collagen‐induced platelet aggregation after combination ART treatment. The aggregation profiles of platelets treated with DTG/3TC, BIC/FTC/TAF, or DMSO in ADP‐evoked aggregation at 3 μM of the agonist exhibited 38.6%, 13.8%, and 11%, respectively, and at 10 μM of the agonist, 61%, 34%, and 24.9%, respectively. At 3 μM TRAP‐6‐evoked platelet activation, the DTG/3TC group demonstrated an 8.3% aggregation, the BIC/FTC/TAF group a 5% aggregation, and the DMSO group a 2.13% aggregation. Lastly, at 1 μg/mL collagen‐induced activation, platelets treated with DTG/3TC, BIC/FTC/TAF, or vehicle displayed 34.3%, 10.3%, and 8% aggregation, respectively. Comparisons between therapeutic drug combinations are displayed in Figure 3.

### Enhanced platelet aggregation was not associated with changes in established markers of platelet activation

**FIGURE 4:**
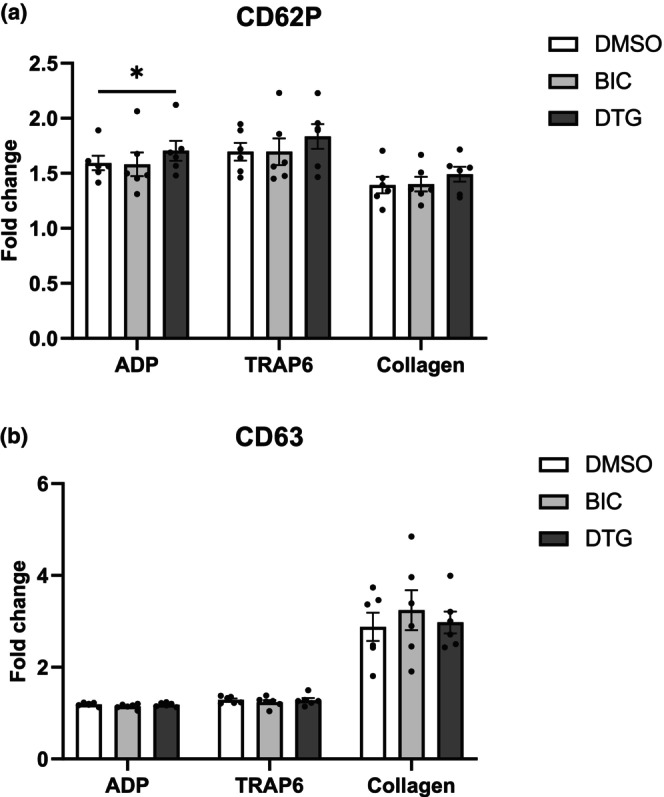
Fold change in CD62P and CD63 levels after in vitro treatment of platelets with bictegravir (BIC) or dolutegravir (DTG). The graph shows (a) the fold change in CD62P (P‐selectin) in platelet‐rich plasma (*N* = 6) treated with BIC, DTG, or vehicle control (dimethylsulfoxide [DMSO]) after 10 μM adenosine 5′‐diphosphate (ADP), 10 μM thrombin receptor activator for peptide 6 (TRAP‐6), or 10 μg/mL collagen activation, in flow cytometry. Graph (b) shows the fold change in CD63. Results from *N* = 6 were analysed with two‐way analysis of variance, and groups were compared using Tukey's multiple comparison test: *p < 0.05.

Degranulation of platelets is an established component of the platelet activation process, leading ultimately to platelet aggregation. Platelet degranulation was examined after treatment with INSTI. CD62P (or P‐selectin) and CD63 are α‐granule and dense‐granule markers, respectively, and were measured by flow cytometry during real‐time platelet activation with 10 μM ADP or TRAP‐6 or 10 μg/mL of collagen. Figure 4 shows no significant differences between treatment groups after TRAP‐6 or collagen‐evoked activation. The fold change of CD63 was not significantly different between INSTI and control groups in all three agonist‐induced platelet activation. In only one instance, the DTG group activated with ADP exhibited an apparently small but statistically significantly higher fold change in CD62P than the DMSO group (*p* = 0.04). This observation was not consistent between agonists despite a broader effect in light‐transmission data. Thus, aggregation responses were not associated with changes in conventional markers of platelet activation, weakening the evidence of a link between drug exposure and platelet activation. We then progressed to studies in people with HIV to explore whether the in vitro effects on platelets were replicated in patients on effective INSTI‐based therapies.

### Pilot study from people living with HIV on BIC/FTC/TAF or DTG/3TC

**FIGURE 5:**
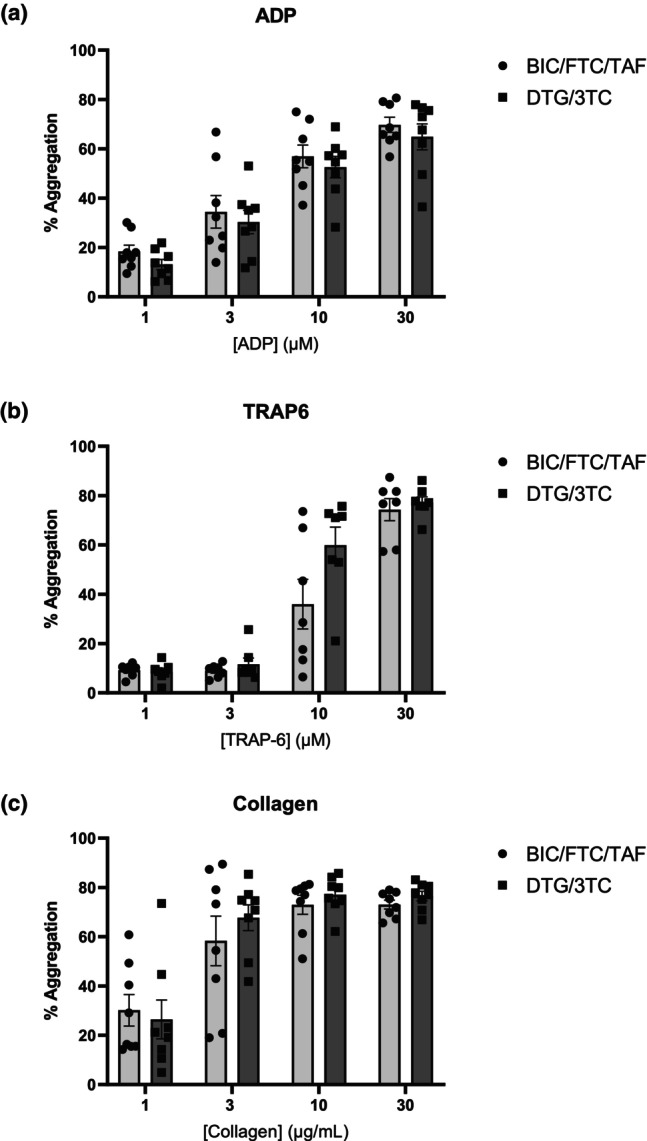
Aggregation profiles of platelets from people living with HIV on bictegravir (BIC)/emtricitabine (FTC)/tenofovir alafenamide fumarate (TAF) or dolutegravir (DTG)/lamivudine (3TC). The figure shows (a) the adenosine 5′‐diphosphate (ADP)‐induced platelet aggregation in platelet‐rich plasma (PRP) from people with HIV, *N* = 8 on BIC/FTC/TAF and *N* = 8 ON DTG/3TC. Row (b) demonstrates thrombin receptor activator for peptide 6 (TRAP‐6)‐induced aggregation in PRP from people with HIV in the two treatment groups, and row (c) the collagen‐induced aggregation. Results are analysed using two‐way analysis of variance, and groups were compared with Tukey's multiple comparison. No statistically significant differences were observed.

Table 1 shows the demographics of the 16 patients recruited to the study, eight from each treatment group. The table demonstrates participant profiles, including age, ethnicity, sex at birth, and co‐medications for every participant individually. All participants had a viral load <50 copies. Figure 5 illustrates platelet aggregation profiles from people with HIV on BIC/FTC/TAF or DTG/3TC. We made comparisons between treatment groups and observed no statistical difference at any data point. Thus, the effects seen when drugs were applied in vitro were not replicated in an ex vivo setting using the same method of laboratory analysis.

**TABLE 1:** Patient demographics of 16 people with HIV recruited in the pilot study receiving BIC/FTC/TAF or DTG/3TC.

Demographic	BIC/FTC/TAF	DTG/3TC
*N* (sample size)	8	8
Age (mean years ± SD)	53.1 (± 5.9)	53.1 (± 5.8)
Ethnicity (n)		
Caucasian	6	5
Black (African)	1	0
Multiple/mixed ethnicity	0	1
Not stated	1	2
Sex at birth (M/F)	8/0	8/0
Viral load <50 copies/mL	8	8
Co‐medications	P1: atorvastatin, perindopril, amlodipine	P9: propecia, minoxidil
	P2: citalopram, acyclovir	P10: atorvastatin, colecalciferol, doxycycline lansoprazole, zoledronic acid
	P3: none	P11: none
	P4: atorvastatin	P12: sertraline
	P5: metformin, amlodipine, ramipril, glitazone	P13: sertraline
	P6: none	P14: none
	P7: atorvastatin	P15: atorvastatin, alendronic acid
	P8: finasteride, escitalopram	P16: vitamin D

Abbreviations: *BIC* bictegravir, *DTG* dolutegravir, *F* female, *FTC* emtricitabine, *M* male, *P* participant, *SD* standard deviation, *TAF* tenofovir alafenamide fumarate, *3TC* lamivudine.

## DISCUSSION

Given the higher risk of developing CVD [1, 2, 4], greater understanding of the underlying causes and treatment of cardiovascular comorbidity in people with HIV is required. There are numerous clinical studies reporting that CVD risk is associated with ART use and other observations of the same [1, 2]; in contrast, other studies have failed to confirm earlier reported associations [3]. Understanding the effects of individual antiretroviral components of ART combinations is important because it potentially allows personalisation of prescribing practice to reduce the detrimental impact of ART on the cardiovascular system. Platelets and endothelial cells are key drivers of thrombotic CVD and vascular disease, and their function can be characterized in great detail. We have previously explored the effects of NRTIs on platelets and cultured endothelial cells in studies involving the application of individual drugs at clinically relevant concentrations [13, 14]. With a big shift towards INSTIs over the last decade [24], and relatively little known about their off‐target effects on cellular drivers of CVD such as platelets, we focused this study on commonly used INSTI‐based regimens BIC/FTC/TAF and DTG/3TC, considering the effects of both individual drugs and therapeutic combinations. We focused on well‐established models of platelet activation and therapeutically relevant drug concentrations. We used both in vitro and ex vivo studies and identified key strengths and limitations of both studies that should be considered when interpreting data comparing off‐target effects of clinically approved therapeutic drugs.

Initial studies focused on light‐transmission aggregometry assays, which are considered the gold standard model of platelet activation [25], correlating well with thrombotic activity in a range of settings from antithrombotic drug development and platelet signalling [26] to validating pollution‐associated CVD risk [27]. We conducted studies in blood from volunteers without HIV and exposed their ART‐naïve PRP to clinical C_max_ of DTG and BIC, alone or in relevant clinical combinations in vitro. We used established methods of acute drug exposure that increase the likelihood of any observed effect being pharmacological in nature and reversible upon switching to an alternative therapeutic [13].

Our results show a significantly higher aggregation profile in DTG‐treated platelets than with BIC or DMSO. This profile was consistent across a range of platelet agonists and could be seen consistently in experimental settings involving both individual application of drugs and therapeutic combinations. Given that platelet aggregometry simply measures light transmission through PRP, we wanted to confirm that observed differences reflected changes in active aggregation rather than either an artefactual effect on light transmission or a passive agglomeration effect leading to changes in light transmission independent of canonical platelet activation pathways [28]. Platelet activation is mediated via α_IIb_β_3_, a transmembrane receptor for fibrinogen that mediates platelet‐to‐platelet aggregation. We demonstrated that DTG‐enhanced light transmission was abolished by the α_IIb_β_3_ antagonist eptifibatide, indicating a α_IIb_β_3_‐mediated effect and ruling out passive agglomeration processes. Although most of the significant changes in platelet aggregation occurred with DTG, we observed that BIC‐treated platelets showed an augmented aggregation response relative to DMSO at 3 μM TRAP‐6. Thus, both DTG and BIC can affect platelet aggregation when applied in vitro. However, whether these changes are limited to in vitro aggregometry or have broader significance remains unclear. We therefore progressed to additional well‐established flow‐cytometric platelet assays.

Platelet aggregation is driven by underlying signalling events involving receptor activation and processes such as degranulation, which drive thrombotic events and contribute to CVD [29]. We therefore explored platelet degranulation via flow‐cytometric analysis of surface expression of CD62P and CD63 as indices of α‐granule and δ‐granule release, respectively. Our data demonstrate an apparently small but statistically significant increase in CD62P release in DTG‐treated platelets after stimulation with ADP. This effect was isolated to one marker and was not seen with other agonists. Thus, although widespread effects of INSTIs on platelet aggregation were measured as light transmission, these effects were not consistently associated with established markers of platelet activation. It is important to highlight that this study explores off‐target effects of drugs that are designed to target viral activity. Their effects on mammalian cells are likely to be non‐specific and may not be related to established mechanistic pathways. Thus, although the platelet assays that we used are well‐established when looking at platelet‐targeting drugs, any effect on an individual assay, even one that is considered the gold standard, should be interpreted with caution, and we recommend that mechanistic studies incorporate a combination of functional assays to confirm that any effect is observed consistently. The absence of an effect on established markers of activation in our study means it is not possible to link any of the drugs under observation with a consistent effect on platelet and therefore cardiovascular function.

Our study involved in vitro application of individual drugs and drug combinations in an experimental design that allowed drugs to be compared with vehicle controls and with each other in platelet samples from an individual donor. For the aggregometry assays, each comparison was made on an individual 96‐well plate. This single‐variable approach does have mechanistic advantages but is distant from the patient setting. A key limitation of our study is that in vitro findings, even if they had been consistent across the assays employed, may not be reflected in people. Acute drug exposure is mechanistically useful but very different from long‐term daily drug intake. Furthermore, studying individual cell types in isolation is mechanistically useful but risks over‐interpretation of effects that have not been confirmed in a real‐world, patient setting. Therefore, whether the observed changes in platelet activation in vitro translate into the development and progression of CVD remains unclear. Ultimately, platelet studies should include analysis involving people, which is time consuming and expensive. Nonetheless, we conducted a pilot study to compare platelet profiles in people living with HIV on BIC/FTC/TAF or DTG/3TC. Comparator groups were roughly matched for basic demographics such as age and sex, although there were differences in comorbidity that we have summarized and are inherent in real‐world settings.

We focused on light‐transmission aggregometry because this was the most affected parameter in our in vitro studies, and we did not see any significant changes between our patient cohorts. Post‐study power calculations were conducted to calculate the appropriate sample size for the clinical study using the online tool OpenEpi (https://www.openepi.com/SampleSize/SSMean.htm). For 80% power and a 95% confidence level, given the ratio of the two treatment populations is 1, the recommended sample size is 182: 91 participants in each treatment group. Data from the 3 μg/mL collagen‐induced platelet aggregation (Figure 5c) were used for this calculation. Thus, our study was under‐powered and should be considered a pilot.

Future platelet studies involving people with HIV should carefully consider the sample sizes required to deliver powered analysis. An alternative approach could be an interventional study in which participants switch their drug regimen. This would permit intra‐individual analysis and a more realistic cohort size. Further mechanistic studies could also involve more physiologically relevant assays incorporating shear flow at physiological and pathological rates as well as assays incorporating multiple cell types and animal studies to explore platelet function and thrombus formation at a whole‐organism level. Future studies should also include activators of other pathways of platelet stimulation linked with inflammatory activation such as CD40 and chemokine ligands because concentrating on a small number of conventional platelet agonists is a limitation of our study. Our focus on DTG and BIC should also be extended to other INSTIs, such as raltegravir and cabotegravir, to explore whether any of the findings in this study indicate an INSTI class effect. It is also important to apply state‐of‐the‐art platelet functional assays in cohorts of people with HIV to explore at a “big data” level how platelet function may be fundamentally altered in people with HIV. Careful consideration of control groups will be required to maximize the linkage of any effect with HIV status.

In summary, BIC and DTG are commonly prescribed INSTIs with a shared mechanism of action to block retroviral integration. The possibility of differing off‐target effects led us, and others [30], to hypothesize that they may have different effects on biological structures such as the cardiovascular system. In this study, we have shown that it is relatively easy to detect differences between different therapeutic reagents using individual assays. However, it is important to work comprehensively across a range of mechanistic assays and to include studies in people before linking observations with clinical significance. In this study, an effect on platelet aggregation using a well‐established assay was not reflected in other in vitro assays or in pilot patient studies. Our study provides useful insight into the design of studies to explore the off‐target effects of drugs used in the treatment of HIV as well as useful reflections on the interpretation of data comparing different antiretrovirals. Further studies involving modelling of human pathophysiology involving people with HIV are recommended.

## FUNDING

This study was partly funded by an investigator‐lead research grant by Gilead Sciences awarded to ME.

## CONFLICT OF INTEREST STATEMENT

ME and MB have received funding and consultancy fees from pharmaceutical companies, including Gilead Sciences, that are not directly related to this study. There are no further conflicts of interest to declare.

## ETHICS STATEMENT

People without HIV participated following consent under Imperial College Research Ethics Committee approval, and people with HIV were recruited and consented at Chelsea & Westminster Hospital NHS Foundation Trust under NHS Research Ethics Committee approval.
